# Choosing Among Randomized Design Families for Implementation Strategy Trials: A Question-Driven Framework with Practical Tools and Exemplars

**DOI:** 10.21203/rs.3.rs-10616678/v1

**Published:** 2026-08-14

**Authors:** Jonah K. Amponsah, Shellie D. Ellis, Felice Resnik

**Affiliations:** Carbone Cancer Center, University of Wisconsin-Madison; Carbone Cancer Center, University of Wisconsin-Madison; University of Wisconsin-Madison

**Keywords:** implementation science, implementation strategies, cluster-randomized trials, stepped-wedge designs, Multiphase Optimization Strategy, SMART, adaptive implementation, experimental design

## Abstract

**Background:**

Implementation science increasingly tests implementation strategies as interventions in their own right, but guidance for choosing among randomized design families remains dispersed across separate methodological literatures. Design selection can become method-driven rather than question-driven, producing trials that do not support the intended implementation decision. We developed a practical framework for selecting randomized designs when the implementation strategy, its components, rollout timing, or sequence is the randomized factor.

**Methods:**

We conducted a focused methodological synthesis updated through May 31, 2026. Because contemporaneous records of the initial exploratory search were unavailable, we conducted a structured verification search using domain-restricted queries targeting PubMed/PubMed Central records and publisher pages. Terms covered implementation strategies, cluster-randomized and stepped-wedge trials, factorial/MOST designs, SMARTs, and adaptive implementation. Sources were classified as completed trials, protocols, methods papers, or boundary examples. We compared design families by question, randomized factor, causal contrast, timing, data requirements, strengths, limitations, and feasibility.

**Results:**

Four complementary design families address four recurring decisions. Parallel and multi-arm cluster-randomized trials compare static implementation strategies. Stepped-wedge cluster-randomized trials evaluate randomized, staged rollout when timing can be assigned, and all clusters are expected to receive the strategy. Factorial experiments within the Multiphase Optimization Strategy identify active, redundant, or interacting components of multicomponent strategies. Sequential multiple assignment randomized trials develop adaptive sequences by re-randomizing sites or providers according to a prespecified intermediate response. The framework is operationalized through a five-step planning sequence, a question-to-design matrix, four design schematics, a worked cluster-trial power sensitivity analysis, and feasibility checks for factorial and adaptive trials.

**Conclusions:**

Randomized implementation strategy trials should begin with the decision the study must support: comparison, rollout, optimization, or adaptation—rather than a preferred method. Explicitly matching that question to the randomized factor, estimand, operational constraints, and realistic sample size, while treating cluster randomized trials, stepped-wedge designs, MOST/factorial designs, and SMARTs as complementary options within a unified framework, can improve rigor, efficiency, and interpretability and clarify when a simpler design is preferable. The accompanying tools provide practical guidance for investigators, implementation scientists, trialists, biostatisticians, funders, and research design cores, including CTSA-supported design and biostatistics cores.

**Contributions to the literature:**

- Existing guides often describe individual trial designs but provide limited help choosing among randomized designs when an implementation strategy itself is assigned.
- This paper organizes four design families around the decision the trial must support: comparison, rollout, component optimization, or adaptation.
- Published trials and protocols illustrate how the randomized factor, unit of assignment, timing, and causal contrast differ across designs.
- A planning sequence, comparative table, design schematics, and worked feasibility calculations help teams justify design choices and identify when a simpler design is preferable.

## Introduction

Implementation science seeks to improve the uptake, fidelity, reach, and sustainment of evidence-based practices in real-world settings. It does so partly by developing and testing implementation strategies, including training, facilitation, audit and feedback, clinical decision support, workflow redesign, and changes in professional roles or organizational infrastructure (1, 2). Randomized experiments are not the only appropriate approach to implementation research, but they remain important when the objective is to estimate the causal effect of an implementation strategy on implementation, service, or clinical outcomes (3–5).

A central boundary condition for this review is the distinction between an evidence-based intervention and an implementation strategy. The evidence-based intervention is the clinical, public health, or service practice being implemented. The implementation strategy is the method used to promote adoption, fidelity, reach, scale-up, or sustainment of that practice. The designs considered here apply when the implementation strategy, a strategy component, rollout timing, or strategy sequence is the randomized factor. Trials in which only the evidence-based clinical intervention is randomized are outside the primary scope.

Choosing a randomized design is not a neutral technical step. A conventional two-arm cluster-randomized trial can compare two static strategies but cannot identify which elements of a strategy bundle are active. A stepped-wedge design can preserve randomization during staged rollout but cannot by itself optimize individual components or specify how support should change for nonresponding sites.

Factorial experiments can estimate component effects, while sequential multiple assignment randomized trials can evaluate adaptive strategy sequences. Beginning with a familiar method rather than the intended decision can therefore yield an underpowered or poorly aligned trial even when the analysis is technically correct.

Existing guidance provides important but fragmented pieces of this decision. Broad reviews cover experimental, quasi-experimental, observational, and qualitative approaches (5–7). Guidance for randomized implementation trials has focused primarily on parallel cluster designs and related conduct and reporting issues (8–10). Strategy catalogs summarize what has been tested and with which outcomes (11, 12). Separate literatures describe optimization through factorial designs and the Multiphase Optimization Strategy (MOST) (13–15), and adaptation through sequential multiple assignment randomized trials (SMARTs) (16, 17). Empirical work also provides reference values for multilevel power planning (18). These contributions are substantial, but investigators still lack a concise framework that links common implementation decisions to randomized design families and real-world exemplars.

The distinction is particularly important as implementation studies become more pragmatic, multicomponent, and embedded within health systems. Hybrid effectiveness-implementation studies may use any of several allocation designs; the hybrid type indicates the relative emphasis on clinical effectiveness and implementation, whereas terms such as cluster-randomized, stepped-wedge, factorial, and SMART describe how assignment occurs (3, 4). Treating these labels as interchangeable obscures both the scientific question and the estimand.

This paper offers a focused, strategy-oriented synthesis of four randomized design families: parallel and multi-arm cluster-randomized trials (CRTs), stepped-wedge cluster-randomized trials (SW-CRTs), factorial experiments used within MOST, and SMARTs. These families are not an exhaustive taxonomy of experimental methods. They were selected because they correspond to four recurring decisions in implementation research: comparing static strategies, evaluating staged rollout, optimizing multicomponent strategies, and adapting support over time. We map each decision to a randomized factor and causal contrast, distinguish completed trials from protocols and boundary examples, and provide practical tools for design justification and early feasibility assessment.

## Methods

### Review design and scope

We conducted a focused methodological synthesis rather than a systematic review or meta-analysis. The purpose was to integrate design logic and practical planning considerations across selected randomized design families, not to estimate pooled effects or exhaustively enumerate every implementation strategy trial. The manuscript was initially developed through purposive identification of foundational methods papers and illustrative trials and was verified and updated through May 31, 2026.

### Literature identification

The initial evidence base was assembled purposively from foundational methods papers, reviews, and illustrative trials. Contemporaneous records of that exploratory search were unavailable. To improve transparency and verify the current exemplar classifications, a structured verification and update search was conducted on May 31, 2026. Eight domain-restricted queries targeting PubMed/PubMed Central records and official publisher pages combined terms for implementation strategies or implementation interventions with design-specific terms (cluster randomized, stepped wedge, rollout, factorial, fractional factorial, Multiphase Optimization Strategy, SMART, sequential multiple assignment randomized trial, adaptive implementation, and adaptive strategy). The searches returned 34 records; after removal of one duplicate, one correction notice, and one metrics-only webpage, 31 unique candidate reports remained. Titles and abstracts were screened, and full records were examined when the randomized factor, report type, or boundary status was unclear. The existing manuscript bibliography served as a seed set. No new formal journal hand-search, citation-index search, direct author contact, or independent dual screening was undertaken during the reconstruction. Complete queries, eligibility rules, result accounting, and candidate-source dispositions are provided in Additional file 1.

### Eligibility and exemplar classification

Eligible English-language sources included: (1) methodological papers that defined, extended, or provided planning guidance for one of the design families; and (2) empirical trials or protocols in which an implementation strategy, strategy component, rollout time, or strategy sequence was randomized. We prioritized sources with sufficiently clear descriptions of the unit of randomization, assignment schedule, primary implementation question, and intended causal contrast. Studies in which only the clinical intervention was randomized were excluded from the exemplar set. Systematic reviews and reporting guidelines were retained as supporting evidence but were not labeled as trial exemplars. The corresponding author directed the search strategy, eligibility decisions, and source classification. ChatGPT was used only for assistance with organizing retrieved records and identifying potential title duplicates; all final inclusion and classification decisions were made by the authors.

Each exemplar was classified as a completed randomized trial, a study protocol, a methods paper, or a boundary example. Boundary examples were retained when they clarified an important application but did not fully satisfy the review boundary. For example, the Broder-Fingert et al. protocol uses factorial randomization to optimize delivery components and therefore illustrates factorial logic, although its randomized components are not exclusively organizational implementation strategies (19). Aydin et al. applied a retrospective, nonrandomized factorial analysis to existing implementation data and is therefore treated as a feasibility proof-of-concept rather than evidence from a randomized implementation trial (20).

### Framework development and practical outputs

For each design family, we charted the primary implementation decision, randomized factor, unit and timing of randomization, primary causal contrast, data structure, principal strengths, limitations, and feasibility considerations. We then organized the families around four question types: comparison, rollout, optimization, and adaptation. The resulting framework was translated into three practical outputs: a five-step planning sequence, a question-to-design selection matrix with labeled exemplars, and schematics showing the randomization logic of each family.

We also developed an illustrative power sensitivity analysis for a parallel CRT with a continuous standardized outcome. The calculation uses the conventional individual-randomized approximation and inflates it by the equal-cluster-size design effect, 1 + (m − 1)ICC. It is intended only as an early screening calculation. Final power analyses should reflect the planned estimand, outcome distribution, unequal cluster sizes, repeated measures, covariate adjustment, small-sample degrees of freedom, attrition, and the intended mixed-model or marginal analysis, preferably through specialized software or simulation.

### Reporting guideline

Reporting of the literature-identification and source-selection procedures was guided by the EQUATOR-listed reporting standards for literature searches and report inclusion criteria. A completed checklist, full verification-search queries, eligibility and classification rules, result accounting, and candidate-source disposition log are provided in Additional file 1.

### Use of generative artificial intelligence

OpenAI’s ChatGPT was used to assist with retrieval organization, title deduplication, and preparation of the reporting checklist. The authors reviewed and are responsible for all substantive content, citations, classifications, calculations, interpretations, and final wording.

## Results

### A question-driven planning sequence

The synthesis identified a consistent decision sequence across design families. Teams first need to define what is being randomized and what implementation decision the trial must support. Only then should they select a design family, specify the estimand and analysis, and assess whether the required number of clusters, periods, or re-randomized units is operationally feasible. Table 1 summarizes this sequence. It is intended as a first-pass planning aid rather than an algorithm or substitute for protocol-specific statistical consultation.

### Parallel and multi-arm cluster-randomized trials

Parallel and multi-arm CRTs are best suited to comparative questions about static implementation strategies delivered at a group level. Clusters such as clinics, hospitals, schools, practices, or care teams are randomized once to two or more strategy conditions. Cluster assignment is often necessary because strategies change shared workflows, supervision, leadership, or electronic systems and would create substantial contamination if individuals within the same organization were assigned to different conditions (9, 10).

The principal causal contrast is typically the average difference in an implementation outcome between strategy conditions during a prespecified follow-up period. Questions include whether facilitation adds benefit to education alone, whether enhanced audit and feedback outperforms standard feedback, or which of several static implementation packages produces greater adoption, fidelity, or reach. Completed exemplars include practice-level strategies for translating self-management support into primary care (21), a multifaceted strategy to increase out-of-office blood pressure monitoring (22), and enhanced audit and feedback to improve uptake of childhood pneumonia policy (23). Systematic review evidence indicates that audit and feedback effects vary across contexts and delivery features, reinforcing the need to specify the exact strategy and comparator (24).

CRTs offer familiar design logic, direct causal interpretation, and compatibility with pragmatic delivery. Their principal constraints are the number of independent clusters, sensitivity to intraclass correlation, imbalance in cluster size, and limited ability to identify active components within a bundled strategy. They are also nonadaptive: clusters continue with their assigned strategy even when early uptake is poor. A hybrid type 3 study may use a parallel CRT, but “hybrid type 3” describes the study focus rather than the randomization structure (3, 4) These constraints motivate other design families when implementation questions involve rollout, optimization, or adaptation over time.

### Stepped-wedge and randomized rollout designs

SW-CRTs randomize the timing or sequence of implementation rather than permanent receipt of the strategy. Clusters begin in a control or usual-support condition and cross to the implementation strategy at assigned time points. This structure is appropriate when staged rollout is operationally necessary, timing can be randomized, repeated outcome measurement is available, and all participating clusters are expected to receive the strategy (25, 26).

Completed implementation exemplars include practice facilitation to improve cardiovascular care (27), practice facilitation to accelerate uptake of a multilevel adherence intervention (28), and facilitation to reduce excessive postoperative antibiotic prophylaxis across children’s hospitals (29). HealthyHearts NYC and the CARE facilitation study provide protocol examples that clarify the sequence and analytic logic of stepped-wedge implementation trials (30, 31). Randomized rollout has also been used to evaluate system-level quality improvement initiatives under rapidly changing conditions (32).

Eventual receipt should not be treated as sufficient ethical justification for a stepped-wedge design. Investigators still need equipoise regarding the timing or effect of the strategy, the ability to randomize rollout order, protection against anticipatory implementation, and an analysis that accounts for secular time trends and within-cluster correlation. Efficiency depends on the number of clusters, periods, cluster-period size, correlation structure, treatment-effect delay, and availability of baseline and time-varying covariates. With few clusters or strong secular change, a stepped-wedge design may be less credible or less powerful than a parallel CRT.

### Factorial experiments and the Multiphase Optimization Strategy

Factorial experiments address component-level questions within multicomponent implementation strategies. Two or more strategy components are randomized simultaneously, allowing estimation of component main effects and prespecified interactions. A full factorial includes every component combination; a fractional factorial deliberately uses a subset of combinations under explicit assumptions about negligible higher-order interactions. MOST provides a broader preparation-optimization-evaluation framework in which a factorial experiment is often used to select an efficient package before definitive evaluation (13–15).

The key question is not simply whether a bundled strategy works, but which components should be retained, removed, or combined under resource constraints. Wright-Hughes et al. used a randomized fractional factorial screening experiment to study six modifications to audit and feedback and to identify potentially important combinations for subsequent real-world evaluation (33). Broder-Fingert et al. provide a protocol example of factorial optimization of intervention-delivery components, which illustrates the design mechanics but sits near the boundary of implementation strategy research (19). Aydin et al. demonstrate a retrospective, nonrandomized application of factorial logic to existing implementation data; it is useful as a feasibility illustration but cannot support randomized causal inference (20).

Balanced factorial designs can be highly efficient for estimating main effects because every randomized unit contributes to each component contrast. They do not necessarily require a larger total sample than a series of separate trials. Their feasibility is instead constrained by the number and expected magnitude of effects, the randomization level, the number of component combinations that must be delivered reliably, and the assumptions required for fractional designs. Interaction effects are often smaller and less predictable than main effects. In cluster-randomized factorial studies, limited cluster counts can also make variance estimation and robustness checks difficult even when patient-level enrollment is large.

### Sequential multiple assignment randomized trials

SMARTs are designed to develop adaptive implementation strategies: prespecified sequences of decision rules that indicate how support should change according to an intermediate response. Sites, providers, or other units are randomized to an initial strategy and assessed at a defined decision point. Responders may continue, while nonresponders are re-randomized to intensified, switched, or augmented support. The design can estimate effects of initial strategies, second-stage options, and embedded adaptive sequences (16, 17).

A completed clustered SMART evaluated adaptive implementation support for school-based cognitive behavioral therapy across 94 high schools (34). The ADEPT protocol describes a clustered SMART in which clinics not responding to Replicating Effective Programs receive additional facilitation (18). Other protocols apply SMART logic to interpreter use and goals-of-care documentation (35, 36). These examples show that the response criterion is part of the intervention: it must be measurable early enough to trigger adaptation, clinically and operationally meaningful, and specified before the trial begins.

SMARTs mirror real-world escalation of implementation support and can answer several sequential questions within one experiment. They also require substantial planning. Power depends on the number of units entering each randomization stage and on the primary estimand. A trial that enrolls many patients but few sites may have inadequate second-stage information if only a small number of sites are re-randomized. When no valid early-response indicator exists, adaptation cannot be implemented consistently, or the primary question concerns a static comparison, a simpler CRT is preferable.

Figure 1 summarizes the randomization logic of the four design families. It illustrates the single-stage assignment used in parallel cluster-randomized trials, randomized crossover timing in stepped-wedge cluster-randomized trials, simultaneous component assignment in factorial experiments, and response-based re-randomization in SMARTs.

CRT, cluster-randomized trial; MOST, Multiphase Optimization Strategy; SMART, sequential multiple assignment randomized trial. In a SW-CRT, C denotes control or usual support and S denotes the implementation strategy.

### Matching implementation questions to design families

Table 2 converts the four design families into a question-to-design matrix. The matrix separates allocation design from hybrid classification and labels whether cited sources are completed trials, protocols, methods papers, or boundary examples. A protocol can be a useful design exemplar without providing outcome evidence; a methods paper can justify a design choice without being a trial.

### Translating a design family into a trial plan

After selecting a design family, teams should translate the label into a protocol-specific causal structure. Table 3 provides a worked stepped-wedge example. The same fields can be completed for any design family and used during consultation among investigators, operational partners, community stakeholders, and statisticians.

### Worked power and feasibility examples

#### Illustrative parallel CRT sensitivity analysis

Consider a two-arm CRT with a continuous primary implementation outcome, equal allocation, expected standardized effect d = 0.30, two-sided alpha = 0.05, 80% power, and 25 observations per cluster. Ignoring clustering, the large-sample approximation requires about 175 observations. Applying the equal-size design effect, 1 + (25 − 1)ICC, gives the early feasibility values in Table 4. The calculation shows why uncertainty in the ICC can change the required cluster count materially even when the expected number of observations per clinic is fixed.

Williams et al. provide empirical effect-size, ICC, and covariate-R^2^ values from 17 multilevel mental health implementation trials covering 53 outcomes and 81 contrasts (20). Those values are useful for sensitivity analysis, but they are not universal constants for implementation research. Investigators should prioritize estimates from the same outcome, setting, and level of randomization and use broader ranges when the available evidence is indirect.

#### Feasibility checks for factorial and SMART designs

For a 2^4^ factorial trial, 16 component combinations are possible. With only 32 clinics, a balanced design averages two clinics per combination. All 32 clinics contribute to each orthogonal main-effect contrast, but sparse replication limits the ability to examine cluster-level heterogeneity, protect against model misspecification, and estimate small interactions. The team may need fewer components, a fractional factorial design with explicit aliasing assumptions, a more proximal outcome, or a different randomization level.

For a clustered SMART enrolling 30 clinics, an expected 40% nonresponse rate would yield approximately 12 clinics eligible for the second-stage randomization, or about six clinics per second-stage option under equal allocation. That may be insufficient for a primary comparison of second-stage strategies. The design may require more first-stage clinics, a response definition that is scientifically defensible and yields enough re-randomized units, pooling across compatible paths, or a simpler two-arm trial. These calculations should be performed before operational commitments are made because the number of independent randomized units, not the number of nested patients, is usually the binding constraint.

#### Sequencing designs across a program of implementation research

The four design families are complementary rather than mutually exclusive. A program may use a factorial experiment during optimization, evaluate the selected package in a parallel or stepped-wedge CRT, and later use a SMART if meaningful heterogeneity in site response remains. The sequence should not be automatic. Each additional stage is justified only when the preceding evidence supports the next decision, the implementation context remains sufficiently comparable, and the required clusters, outcomes, and operational resources are available.

## Discussion

This review integrates four randomized design families around the implementation decision each is best suited to support. Its central contribution is not a new statistical design but a practical matching framework: implementation question → randomized factor → design family → causal contrast → feasibility assessment. This ordering makes explicit that a trial can be randomized yet still be poorly aligned with its intended implementation decision.

The framework also clarifies conceptual distinctions that are often blurred in planning discussions. An implementation strategy is distinct from the evidence-based intervention it is intended to support. A hybrid type describes the relative emphasis placed on effectiveness and implementation aims, whereas CRT, SW-CRT, factorial, and SMART describe allocation and timing. MOST is an engineering-inspired optimization framework that may use a factorial experiment; a factorial design is not itself synonymous with the full MOST process. These distinctions improve the interpretability of protocols, grant applications, and published reports.

Implementation frameworks and strategy taxonomies can strengthen, but do not determine, experimental design choice. EPIS can locate decisions across exploration, preparation, implementation, and sustainment phases (39). CFIR can identify multilevel determinants that motivate strategy components (40). ERIC can support precise specification of candidate strategies (12). RE-AIM can help define reach, adoption, implementation, and maintenance outcomes (41). These frameworks inform what should be implemented, targeted, and measured; the randomized design then determines which causal contrast can be estimated.

The practical tools have implications for investigators, implementation scientists, trialists, biostatisticians, funders, and research design cores. The five-step sequence encourages teams to define the randomized factor and estimand before negotiating operational details. The matrix prevents a protocol from labeling a study “hybrid” without specifying the allocation design. The schematics create a shared visual language for multidisciplinary teams. The worked examples expose feasibility problems early, when the strategy, response definition, or number of components can still be revised.

Optimization and adaptive designs should be considered when the implementation decision genuinely concerns components or sequences, not because methodological novelty is desirable. A well-powered two-arm CRT is preferable to an under-replicated factorial trial when the immediate decision is whether to adopt one of two packages. Likewise, a SMART is inappropriate when the intermediate response cannot be measured reliably or when there are too few units for second-stage contrasts. Complexity is justified only when it produces information that a simpler design cannot provide.

This synthesis has limitations. It was focused and purposive rather than systematic, and it does not exhaustively catalog randomized implementation trials. Selection of exemplars favored clarity of design logic, which may underrepresent less explicitly reported studies. Empirical applications are uneven across design families; completed clustered SMARTs and randomized factorial implementation experiments remain less common than parallel CRTs. Several useful sources are protocols and therefore illustrate planned design rather than completed execution. Most examples come from health care, mental health, primary care, or school settings, and transfer to other service sectors requires contextual judgment.

The early power examples are intentionally simplified. They do not replace design-specific methods for SW-CRTs, factorial trials, or SMARTs, and the empirical parameters from Williams et al. arise from mental health implementation trials (18). Future work should expand repositories of ICCs, effect sizes, response rates, cluster attrition, cost, and treatment-effect timing across implementation settings. More consistent reporting of the randomized factor, primary estimand, design rationale, and alternatives considered would make such cumulative learning possible.

## Conclusions

Randomized implementation strategy trials should be selected by starting with the decision the study must support. Parallel and multi-arm CRTs compare static strategies; SW-CRTs evaluate randomized staged rollout; factorial experiments within MOST optimize components; and SMARTs develop adaptive sequences of support. These are complementary tools, not interchangeable labels.

A defensible design justification should identify the implementation strategy, randomized factor, unit and timing of assignment, primary estimand, required data structure, and realistic number of independent randomized units. Matching these elements before launch can reduce underpowered or uninterpretable trials and strengthen the cumulative evidence base for implementation strategies.

## Supplementary Material

Supplementary Files

This is a list of supplementary files associated with this preprint. Click to download.

- Additionalfile1.docx

## Figures and Tables

**Figure 1:**
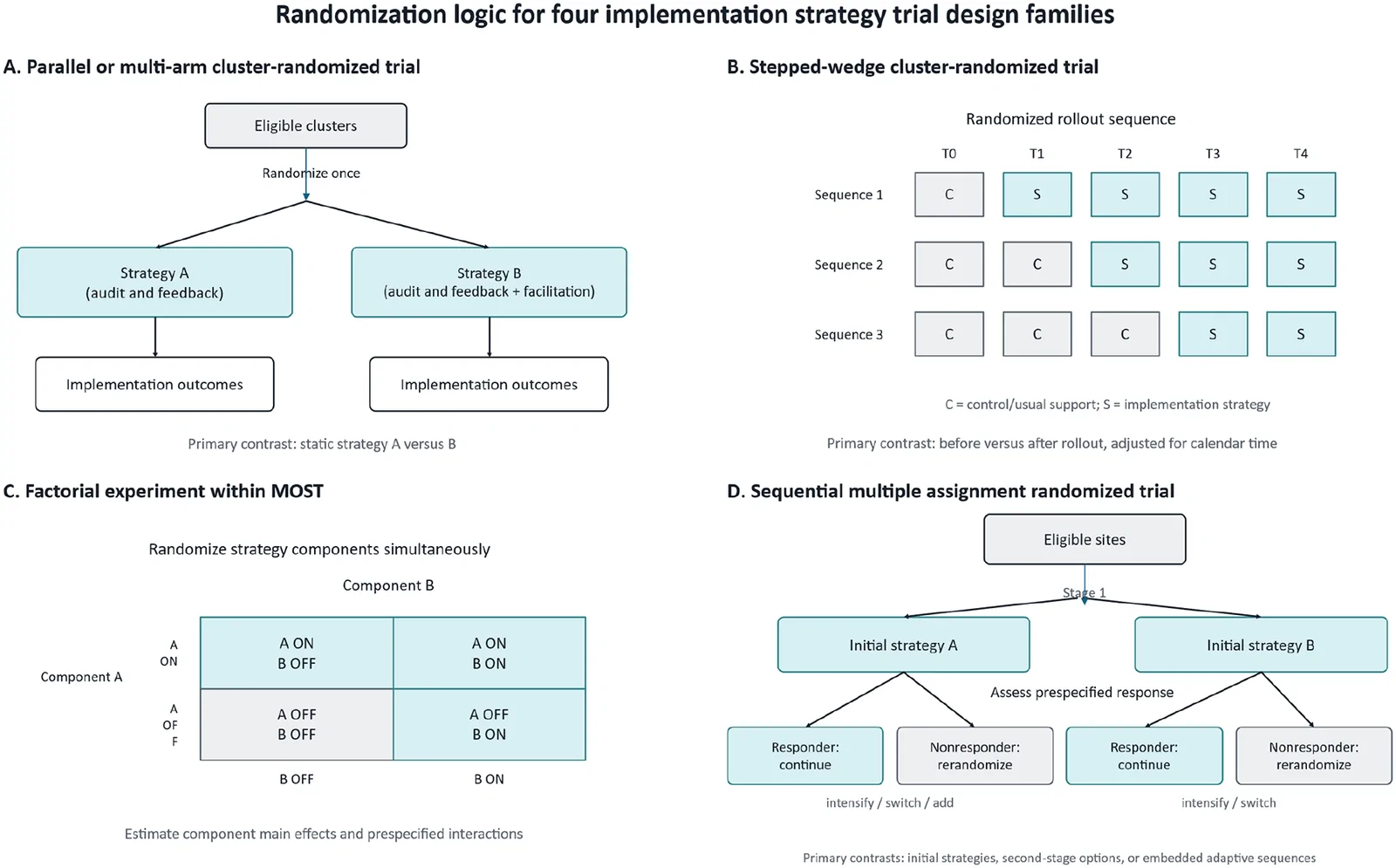
Randomization logic for four implementation strategy trial design families CRT, cluster-randomized trial; MOST, Multiphase Optimization Strategy; SMART, sequential multiple assignment randomized trial. In a SW-CRT, C denotes control or usual support and S denotes the implementation strategy

**Table 1:** Five-step planning sequence for randomized implementation strategy trials

Step	Required question	Planning output
**1. Define the strategy**	Is the randomized factor a whole implementation strategy, one or more components, rollout timing, or a sequence of supports?	A clearly specified implementation strategy distinct from the evidence-based intervention.
**2. State the decision**	Must the study compare static strategies, evaluate staged rollout, optimize components, or adapt support for nonresponse?	One primary implementation question and a decision the result will inform.
**3. Match the design**	Which design family produces the required assignment structure and causal contrast?	A justified choice among CRT, SW-CRT, factorial/MOST, SMART, or a simpler alternative.
**4. Specify the estimand**	What contrast, population, time point, and outcome define the primary effect?	A primary estimand aligned with the randomization and analysis model.
**5. Test feasibility**	Are cluster count, periods, response rates, staffing, data frequency, cost, and timelines adequate?	Sensitivity analyses and a documented reason for rejecting simpler or more complex designs.

**Table 2:** Matching implementation questions to randomized design families and selected sources

Primary implementation question	Design family	Why and when it fits	Selected sources and exemplars
Which static implementation strategy produces greater adoption, fidelity, reach, or sustainment?	Parallel or multi-arm CRT	Randomize clusters once and directly compare prespecified strategy conditions while limiting contamination.	Dickinson et al. (trial) (21); Kronish et al. (trial) (22); Ayieko et al. (trial) (23).
Does adding facilitation or another component improve outcomes beyond standard support?	Parallel or multi-arm CRT; may be conducted as a hybrid type 3 study	Tests the incremental value of an enhanced static package. Hybrid type describes study emphasis, not allocation.	Dickinson et al. (trial) (21); Baffsky et al. (protocol) (37); Curran et al. (hybrid methods) (3).
How can a strategy be rolled out across all sites while retaining randomized evaluation?	SW-CRT or another randomized rollout design	Randomize rollout timing when timing is assignable, repeated measurement is available, and secular trends can be modeled.	Liddy et al., Schoenthaler et al., and McKay et al (trials) (27–29); Shelley et al. and O’Grady et al. (protocols) (30, 31).
Which components of a multicomponent strategy are active, redundant, or interacting?	Full or fractional factorial experiment, often within MOST	Randomize components simultaneously to estimate component effects and selected interactions before definitive evaluation.	Wright-Hughes et al. (completed factorial experiment) (33); Szeszulski and Guastaferro (methods) (13); Broder-Fingert et al. (protocol/boundary example) (19).
How should support change for sites that do not initially respond?	SMART or clustered SMART	Use a prespecified response measure and re-randomize eligible units to compare adaptive sequences.	Smith et al. (completed clustered SMART) (34); Kilbourne et al., Lion et al., and Bekelman et al. (protocols) (35, 36, 38).
How should low- and high-intensity supports be sequenced while considering cost?	SMART with prospective economic evaluation	Compares escalation, augmentation, switching, or continuation rules and can attach costs to embedded sequences.	Kilbourne et al. and Lion et al. (protocols) (36, 38); implementation economic methods should be prespecified.
How should an optimized package be evaluated under routine conditions?	Parallel CRT or SW-CRT; potentially a hybrid type 2 or 3 study	Evaluate the selected package using an allocation design suited to the operational setting. Hybrid classification states the relative study emphasis.	Guastaferro et al. and Szeszulski and Guastaferro (optimization methods) (13, 14); use a CRT or SW-CRT exemplar appropriate to the setting.
Are the available clusters, periods, or second-stage units sufficient?	Cross-cutting feasibility assessment; pilot or simpler design when needed	Use context-specific sensitivity analyses. Do not choose a complex design that cannot estimate its primary contrast reliably.	Williams et al. (empirical multilevel parameters from mental health trials) (18); design-specific statistical consultation.

Hybrid type and allocation design are separate classifications. Protocols illustrate design logic but do not provide completed outcome evidence. Boundary examples do not fully meet the review condition that the implementation strategy be randomized.

**Table 3:** Worked translation of a stepped-wedge design family into a trial plan

Design element	Illustrative stepped-wedge implementation strategy trial
**Primary question**	What is the effect of introducing practice facilitation during a randomized system-wide rollout?
**Unit and factor randomized**	Clinics are randomized to rollout sequence; timing of facilitation is the randomized factor.
**Primary estimand**	Average difference in the prespecified implementation outcome attributable to facilitation over a defined exposure period, conditional on the chosen model for calendar time.
**Measurement schedule**	Comparable outcome measurement in every cluster-period before and after crossover, with explicit handling of transition or wash-in periods.
**Key assumptions**	No unmeasured intervention changes synchronized with rollout sequence; no substantial anticipatory uptake; adequate model for secular trends and correlation.
**Feasibility checks**	Number of clusters and sequences, cluster-period size, ICC and correlation decay, data availability, facilitator capacity, rollout duration, and cluster attrition.
**Analysis implications**	Account for clustering, calendar time, repeated cluster-period observations, unequal sizes, and small numbers of clusters; define whether the effect is immediate or exposure-time dependent.
**Alternative considered**	Use a parallel CRT if simultaneous delivery is feasible and time confounding or prolonged rollout would threaten validity.

**Table 4:** Illustrative early power sensitivity analysis for a two-arm parallel CRT

ICC	Design effect	Approximate observations	Clusters before loss	Clusters allowing 15% loss
0.05	2.20	384	16 (8/arm)	20 (10/arm)
0.10	3.40	594	24 (12/arm)	30 (15/arm)
0.15	4.60	803	34 (17/arm)	40 (20/arm)

Illustrative calculation only. Values use n = 2(z0.975 + z0.80) ^2^ /d ^2^ and the equal-cluster-size design effect. Cluster counts are rounded to preserve equal allocation. The calculation omits small-sample corrections, unequal cluster sizes, repeated measures, covariate adjustment, and outcome-specific modeling.

## Data Availability

No datasets were generated or analyzed for this methodological synthesis. All sources used to develop the framework are cited in the reference list.

## Abbreviations

CFIR: Consolidated Framework for Implementation Research
CRT: cluster-randomized trial
CTSA: Clinical and Translational Science Award
EBI: evidence-based intervention
EPIS: Exploration, Preparation, Implementation, Sustainment
ERIC: Expert Recommendations for Implementing Change
ICC: intraclass correlation coefficient
MOST: Multiphase Optimization Strategy
RE-AIM: Reach, Effectiveness, Adoption, Implementation, Maintenance
SMART: sequential multiple assignment randomized trial
SW-CRT: stepped-wedge cluster-randomized trial
